# Predictive value of atherogenic index of plasma in combination with diagonal earlobe crease in coronary heart disease

**DOI:** 10.3389/fcvm.2025.1632009

**Published:** 2025-08-06

**Authors:** Ruoling Guo, Mingliang Sun, Huihui Yang, Jie Dou, Jie Gao, Tong Liu, Fei Cheng, Donglei Luo

**Affiliations:** ^1^ Department of Chengde Medical University, Chengde, Hebei, People’s Republic of China; ^2^ Department of Emergency, Handan City First Hospital, Handan, Hebei, People’s Republic of China; ^3^ Department of Tianjin Key Laboratory of Ionic-Molecular Function of Cardiovascular Disease, Department of Cardiology, Tianjin Institute of Cardiology, Second Hospital of Tianjin Medical University, Tianjin, People’s Republic of China; ^4^ Department of Neurology, Chengde Central Hospital/Second Clinical College of Chengde Medical University, Chengde, Hebei, China; ^5^ Department of Cardiology, Chengde Central Hospital/Second Clinical College of Chengde Medical University, Chengde, Hebei, China

**Keywords:** coronary heart disease, atherogenic index of plasma, diagonal earlobe crease, predict, model

## Abstract

**Background:**

The atherogenic index of plasma (AIP) and diagonal earlobe crease (DELC) are correlated with the incidence of coronary heart disease (CHD). Nevertheless, the role of AIP and DELC in the characterization and prediction of CHD remains underexplored.

**Methods:**

This study enrolled a total of 1,378 patients suspected to be indicative of CHD, all of whom were admitted to Chengde Central Hospital between September 2021 and August 2024. Based on coronary angiography (CAG), the cohort was stratified into two groups: the CHD group (*n* = 1,071) and the non-CHD group (*n* = 307). Multivariate logistic analysis was used to analyze the interplay of AIP, DELC, and CHD. The predictive value of AIP and DELC for CHD was evaluated by nomogram, calibration curves, and receiver operating characteristic (ROC).

**Results:**

The results showed that AIP and DELC were positively correlated with CHD. Age, gender, hypertension, diabetes mellitus (DM), aspartate aminotransferase (AST), AIP, creatinine (CR), and DELC were independent risk factors for CHD. AIP was correlated with the Gensini score, indicating that as AIP levels rise, the severity of CHD intensifies. A progressive increase in DELC positivity was observed with higher Gensini scores. The diagnostic model for CHD was constructed based on the above risk factors. The area under the curve (AUC) for the integrated application of AIP and DELC was 0.702, highlighting the model's considerable diagnostic efficacy in the identification of CHD.

**Conclusions:**

AIP and DELC were independent risk factors for CHD. The combination of the two factors exhibited great predictive value for the diagnosis of CHD.

## Introduction

Coronary Heart Disease (CHD) is a leading cause of global morbidity and mortality (1). In China alone, approximately 11 million individuals are affected by CHD, presenting a significant public health challenge (2). The pathophysiology of CHD is primarily based on atherosclerosis (3), which can lead to myocardial hypoxia, ischemia, and necrosis when blood vessels become sufficiently narrowed. Coronary angiography (CAG) is universally acknowledged as the definitive diagnostic tool for CHD (4), but it has the disadvantages of invasiveness, high cost, and long hospitalization time, which may be challenging for some patients to endure. In addition, complications during and after the procedure cannot be ignored. Therefore, it is necessary to further find methods that can rapidly identify CHD for effective primary prevention of CHD.

Dyslipidemia is a well-established risk factor for cardiovascular disease and a primary driving force in the development and progression of CHD (5). Studies have demonstrated that a strong correlation between the prevalence of CHD and elevated triglyceride (TG) levels in conjunction with reduced high-density lipoprotein cholesterol (HDL-C) levels (6). The plasma atherogenic index (AIP), defined as the logarithm of the molar ratio of TG to HDL-C, has been identified as an independent risk factor for CHD (7). The underlying mechanism involves the propensity of small, dense low-density lipoprotein (sLDL) particles to undergo oxidative modification, a process that significantly contributes to the development of atherosclerotic plaques (8). Given the established correlation between AIP and sLDL levels (9), AIP can serve as a surrogate marker for the risk of CHD occurrence and progression. Numerous studies have substantiated the role of AIP as a pivotal biomarker for predicting atherosclerosis and CHD (10, 11). Its prognostic value has been well-documented, as evidenced by research indicating a pronounced correlation between elevated AIP levels and an augmented risk of cardiovascular events. For instance, a study examining young adults aged 18–22 years demonstrated that higher AIP values were significantly associated with a greater susceptibility to developing CHD (12). Furthermore, a seminal study by Won et al. (13). (2021) utilized coronary computed tomography angiography (CCTA) to investigate the association between AIP levels and atherosclerotic pathology. Their findings underscored AIP as an independent and robust predictor of atherosclerosis, indicating that it may serve as a more reliable marker for disease progression than traditional cardiovascular risk factors.

In recent years, diagonal earlobe crease (DELC) has been repeatedly demonstrated to be associated with CHD. As a readily identifiable clinical marker, DELC has shown promise in identifying atrisk individuals. In 1973, Frank initially introducted the concept of DELC in the New England Journal of Medicine and subsequently validated the correlation between DELC and CHD (14). Frank's sign is a DELC, which is a wrinkle that extends 45° backward from the tragus to the auricle; it is hypothesized to be a predictor of atherosclerotic disease (15). This phenomenon is closely associated with CHD and atherosclerosis. In patients with CHD, atherosclerosis leads to vascular narrowing or occlusion, resulting in reduced blood flow to the earlobe. This, in turn, affects the tissue elasticity of the earlobe and potentially causes local ischemia and a reduction in the subdermal matrix of the earlobe (16). Consequently, DELC is considered to be the ischemia of the earlobe and the changes in matrix components caused by atherosclerosis (17, 18). A retrospective study, which involved autopsies of CHD patients, demonstrated a robust association between the presence of earlobe creases and the occurrence of coronary atherosclerosis (19). Additionally, a study involving 200 participants revealed markedly higher DELC levels in the CHD group in comparison with the control group (OR = 5.63, 95% CI: 2.91–10.93), further reinforcing the strong association between DELC and CHD (20).

Both the AIP and DELC are recognized as risk factors for CHD. Despite considerable advances in cardiovascular research, there remains a paucity of studies investigating the synergistic effect of AIP and DELC in predicting the onset of CHD. Therefore, this study seeks to explore the correlation between AIP, DELC, and CHD, and to further explore the predictive value of their combined assessment for the incidence of CHD.

## Material and methods

### Study subjects

**Figure 1:**
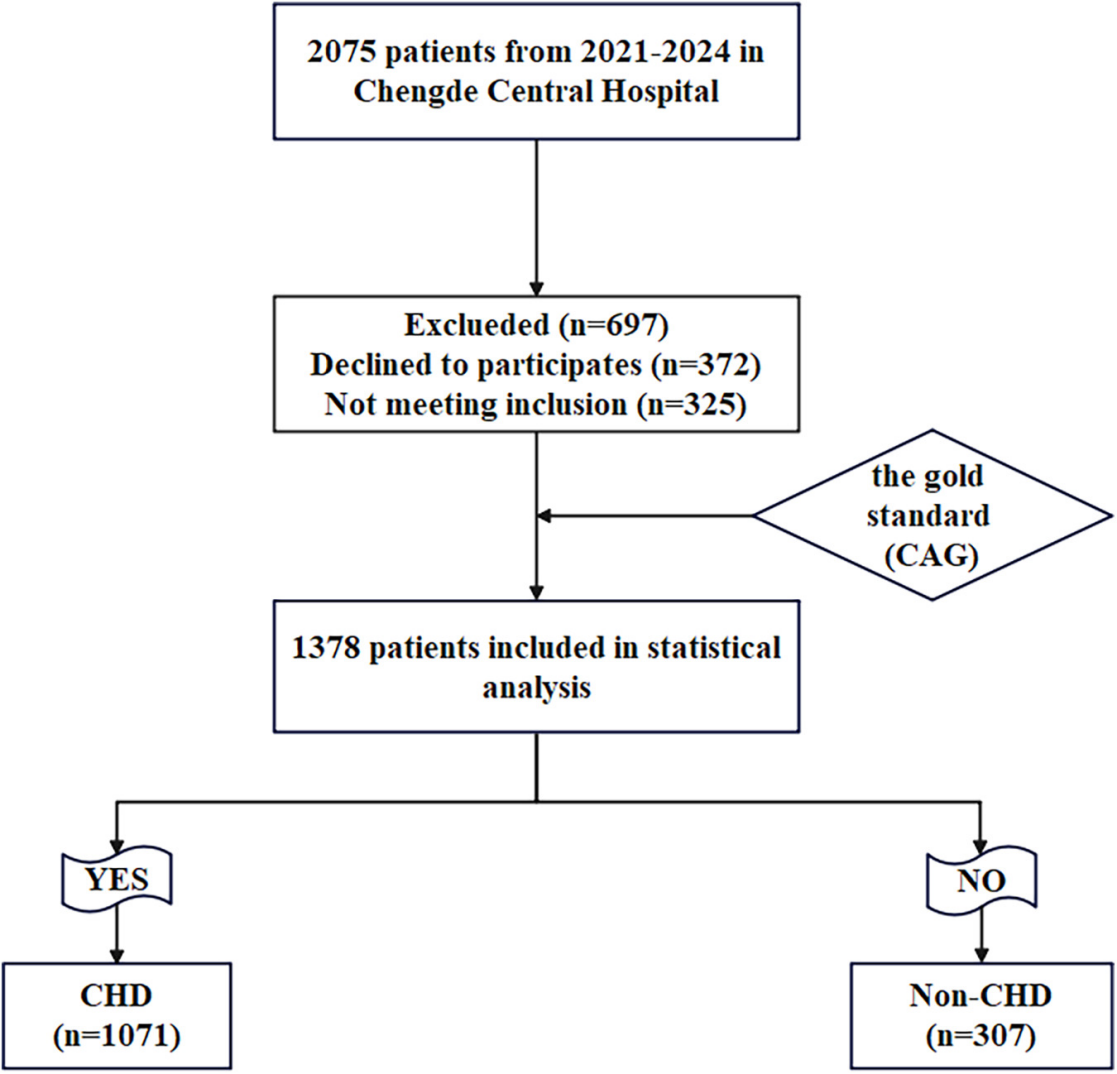
Flow-diagram illustrating patient flow in the study.

This study enrolled 1,378 patients presenting with chest pain and a clinical suspicion of CHD, all of whom subsequently underwent CAG at Chengde Central Hospital between September 2021 and August 2024. According to CAG results, the patients were classified into CHD cohort and Non-CHD cohort. (Figure 1).

### Inclusion and exclusion criteria

Inclusion criteria: (1) Patients older than 18 years old with chest pain and tightness who agreed to CAG; (2) Patients with complete key data, such as TG, HDL-C and DELC.

Exclusion criteria: (1) Previous reperfusion therapy [including thrombolytic therapy, percutaneous transluminal coronary intervention (PCI), or coronary artery bypass grafting (CABG)]; (2) Previous valvulopathy or severe heart failure [New York Heart Association (NYHA) classification ≥2]; (3) Previous severe renal disease or liver failure; (4) Patients with contraindications for CAG; (5) Patients with earrings or ear piercings, or those with previous injuries.

### Data collection

The collected baseline patient data includes: (1) demographic characteristics: age, gender, height, weight, calculated body mass index (BMI) = weight (kg)/height (m)^2^ (kg/m^2^), diagonal earlobe crease (DELC); (2) past medical history: smoking, drinking, hypertension, diabetes mellitus (DM); (3) echocardiography: left ventricular ejection fraction (LVEF); (4) vital signs: diastolic blood pressure (DBP), systolic blood pressure (SBP), heart rate (HR); (5) the first laboratory indicators after admission (morning fasting venous blood drawn upon admission): white blood cells (WBC), hemoglobin (Hb), platelets (PLT), red blood cells (RBC), thyroid-stimulating hormone (TSH), high-sensitivity troponin I (hsTNI), aspartate aminotransferase (AST), triglyceride (TG), creatinine (CR), uric acid (UA), apolipoprotein B (APO-B), lactate dehydrogenase (LDH), glucose (Glu), total cholesterol (TC), lipoprotein (a) [Lp(a)], cystatin C (CysC), low-density lipoprotein (LDL-C), high-density lipoprotein (HDL-C), AIP = 1og(TG/HDL-C).

### Relevant definitions

CHD was identified by the presence of stenosis of 50% or greater in the lumen of at least one principal coronary artery or its branches, as evaluated via CAG (21). CAG was conducted by skilled cardiologists, with the findings subsequently assessed by two independent interventional specialists, both of whom had no conflicts of interest pertaining to the study. In the event of a disagreement, a third physician was consulted. The severity of coronary artery disease in patients with CHD was appraised using the Gensini score (GS) system, which was refined by the American Heart Association (22). Based on the tertiles of the Gensini score, the CHD patient cohort were categorized into three groups: low GS group (<20), medium GS group (20–48), and high GS group (>48) (23, 24). As indicated by extant literature, there is a correlation between GS scores and multivessel disease, with higher Gensini scores corresponding to more severe CAD (25).

AIP is the logarithmic transformation of the ratio between TG and HDL-C [(AIP = 1og(TG/HDL-C)] (7).

DELC: Under the natural light source, the earlobe folds of the patient were observed. DELC was defined as the length of >2/3 of the total earlobe, and DELC was excluded when the length was <2/3 of the total earlobe (26).

### Sample size calculation

The sample size was calculated based on the area under the ROC curve for the model, with all computations performed using R software, version 4.4.1. The risk model demonstrated an AUC of 0.702 for predicting CHD in this study, with a test power (1-β) of 0.9 and a significance level (α) set at 0.05. The ratio of non-CHD to CHD in this study was 1:3. The results indicated that at least 259 patients were needed in the non-CHD group and at least 777 patients in the CHD group. Considering a sample failure rate of 10%, a total of at least 1,140 cases was necessary for this study.

### Data quality and missing data

The data were thoroughly examined for completeness and consistency. All missing values were determined to be Missing Completely at Random (MCAR), and the proportion of missing data was less than 10% of the total sample size. Any inconsistencies or incomplete entries were addressed and rectified accordingly. For cases with substantial missing data, the direct deletion method was employed. For continuous measurement variables with fully observed independent variables, the SPSS regression imputation method was applied. For count data with complete independent variables, the modal imputation technique was utilized.

### Statistical analysis

Quantitative data were evaluated for normality using the Shapiro–Wilk test. Data conforming a normal distribution were presented as mean ± standard deviation (SD) and subjected to analysis via the independent samples *t*-test. In contrast, data that were not normally distributed were expressed as median (Q1, Q3) and analyzed using the Mann–Whitney *U* test. Categorical variables were reported as percentages (%) and subjected to analysis via the chi-square (*χ*²) test. The comparisons of AIP levels in CHD patients with different GS groups should be performed by applying ANOVA with a *post hoc* test. Due to the unequal variances among the groups, the Games-Howell *post hoc* test was employed to conduct multiple comparisons, ensuring the accurate identification of pairwise group differences. The DELC comparison was conducted using the chi-square test. Logistic regression analysis was employed to identify potential influencing factors, with the odds ratio (OR) and 95% confidence interval (CI). In univariate logistic regression, *P* < 0.05 was used as the selection criterion for inclusion in the multivariate regression model, which adjusted for covariates such as age, sex, hypertension, diabetes, smoking, SBP, LVEF, AST, CR, and CysC, calculating the adjusted OR and 95% CI. The multivariate logistic regression model was visualized, and corresponding nomograms were created. The diagnostic performance of the model was evaluated through the ROC curve, with the AUC serving as a metric for evaluating its discriminative ability. The variance inflation factor (VIF) was calculated to assess the presence of multicollinearity among the predictor variables. Using the Hosmer-Lemeshow test and the curve of calibration, the predictive ability of the AIP combined with DELC for CHD was evaluated. *P* > 0.05 indicated strong model calibration and good fit. To enhance the reliability of the model, a Decision Curve Analysis (DCA) was performed to assess its potential in promoting clinical decision-making. *P* < 0.05 was considered indicative of statistical significance. Statistical analyses in the present study were conducted using SPSS software version 27.0 (IBM Corporation, Armonk, NY, USA), while graphical representations were constructed utilizing R software version 4.4.1 and GraphPad Prism 8.0.

## Results

### Clinical baseline data comparisons

A total of 325 cases were excluded based on the established exclusion criteria, leaving 1,378 cases as the final study cohort. This comprised 1,071 patients with CHD in the case cohort and 307 controls without CHD in the control group. Noteworthy differences were observed between the non-CHD and CHD groups with respect to several clinical parameters, including age, gender, hypertension, DM, smoking, SBP, LVEF, WBC, Hb, hsTNI, AST, CR, LDH, Glu, TG, HDL-C, AIP, Lp(a), CysC and DELC between the Non-CHD group and the CHD cohort (*P* < 0.05). No statistically significant differences were identified across the other indicators (*P* > 0.05) (Table 1).

**Table 1:** Baseline characteristics with and without CHD.

Variables	Non-CHD	CHD	*t/χ^2^/Z*	*P* value
	(*n* = 307)	(*n* = 1,071)		
Age (years)	58.88 ± 8.89	61.17 ± 9.50	−3.764	0.000
Male	129 (42.60)	655 (60.90)	32.475	0.000
Female	174 (57.40)	420 (39.10)	32.475	0.000
BMI (kg/m^2^)	25.48 ± 3.38	25.54 ± 3.55	−0.261	0.794
Hypertension	166 (54.10)	710 (66.30)	15.389	0.000
DM	56 (18.20)	322 (30.10)	16.759	0.000
Smoking	101 (32.90)	520 (48.60)	23.617	0.000
Drinking	90 (29.30)	367 (34.30)	2.639	0.104
Heart rate (beats/min)	77.21 ± 12.65	76.42 ± 13.12	0.942	0.346
SBP (mmHg)	135.07 ± 18.71	138.36 ± 19.89	−2.586	0.010
DBP (mmHg)	83 (75,90)	82 (74,91)	−0.692	0.489
LVEF (%)	57.92 ± 4.09	57.03 ± 5.23	2.730	0.006
WBC (×10^9^/L)	6.23 ± 1.80	6.70 ± 2.08	−3.654	0.000
RBC (×10^12^/L)	4.54 ± 0.46	4.59 ± 0.55	−1.513	0.130
Hb (g/L)	140.27 ± 14.55	142.47 ± 15.25	−2.254	0.024
PLT (×10^9^/L)	214.38 ± 56.64	214.36 ± 55.71	0.007	0.995
TSH (mmol/L)	2.83 ± 1.76	3.12 ± 2.92	−1.672	0.095
hsTNI (ng/ml)	0.42 ± 2.20	2.40 ± 7.81	−4.387	0.000
AST (U/L)	23.72 ± 17.24	29.61 ± 39.84	−2.521	0.012
UA (umol/L)	317.92 ± 95.96	325.09 ± 91.01	−1.202	0.229
CR (umol/L)	62.29 ± 13.34	68.92 ± 18.18	−5.942	0.000
LDH (U/L)	183.05 ± 73.44	209.96 ± 173.79	−2.646	0.008
Glu (mmol/L)	5.69 ± 1.38	6.22 ± 2.16	−4.110	0.000
TG (mmol/L)	1.72 ± 1.08	2.02 ± 1.60	−3.147	0.002
TC (mmol/L)	4.22 ± 1.02	4.30 ± 1.10	−1.231	0.218
HDL-C (mmol/L)	1.23 ± 0.38	1.12 ± 0.28	5.400	0.000
LDL-C (mmol/L)	2.23 ± 0.76	2.32 ± 0.82	−1.791	0.073
AIP	0.10 ± 0.28	0.19 ± 0.30	−4.528	0.000
APO-B (g/L)	0.90 ± 0.81	0.93 ± 0.51	−0.812	0.417
Lp(a) (mg/dl)	24.30 ± 21.13	29.02 ± 27.47	−2.786	0.005
CysC (mg/L)	0.94 ± 0.17	1.01 ± 0.22	−5.391	0.000
hypertrilipidemia	43 (14.00)	184 (17.20)	1.747	0.186
DELC	195 (63.50)	818 (76.40)	20.263	0.000

BMI, body mass index; DM, diabetes mellitus; SBP, systolic blood pressure; DBP, diastolic blood pressure; LVEF, left ventricular ejection fraction; WBC, white blood cells; RBC, red blood cells; Hb, hemoglobin; PLT, platelets; TSH, thyroid-stimulating hormone; hsTNI, high-sensitivity troponin I; AST, aspartate aminotransferase; UA, uric acid; LDH, lactate lehydrogenase; Glu, glucose; TG, triglycerides; TC, total cholesterol; HDL-C, high density lipoprotein cholesterol; LDL-C, low density lipoprotein cholesterol; Lp(a), lipoprotein (a); AIP, atherogenic index of plasma; APO-B, apolipoprotein B; Cys-C, cystatin C; DELC, diagonal earlobe crease.

### Analysis of AIP, DELC and Gensini score in patients with CHD

**Figure 2:**
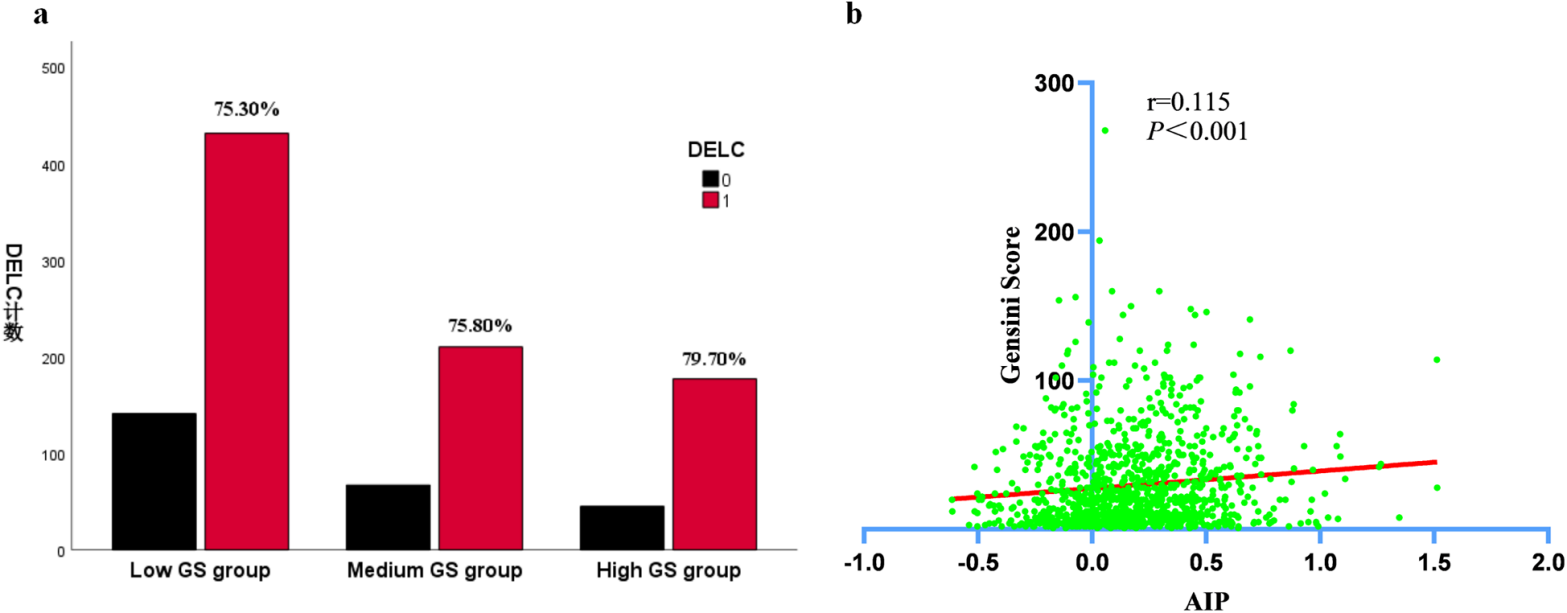
Comparisons of DELC ratio and AIP levels in CHD patients with different GS group. **(a)** Comparisons of DELC ratio in CHD patients with different GS group; **(b)** Comparisons of AIP levels in CHD patients with different GS group.

To explore the interplay between CHD patients and the Gensini score, we conducted an analysis of the AIP and DELC parameters. Due to the unequal variances among the groups, the Games-Howell *post hoc* test was employed to conduct multiple comparisons, ensuring the accurate identification of pairwise group differences. The results confirmed that the concentrations of AIP in the medium and high GS groups were substantially elevated in comparison to those in the low GS group (*P* < 0.05). We used Pearson's correlation to evaluate the correlation between AIP and Gensini score. AIP exhibited a significant positive connection with the Gensini score (*r* = 0.115, *P* < 0.001). As shown in the Figure 2b, the intensity of coronary artery lesions in CHD patients was aggravated with the increase of AIP levels. As the Gensini score increased, the proportion of DELC positivity in each group also rose progressively (Figure 2a). (Table 2, Figure 2).

**Table 2:** Comparisons of DELC and AIP levels in CHD patients with different GS group.

Variables	Low GS group	Medium GS group	High GS group	*P_a_*	*P_b_*	*P_c_*
	(*n* = 572)	(*n* = 277)	(*n* = 222)			
DELC	431 (75.30)	210 (75.80)	177 (79.70)	0.413		
AIP	0.15 ± 0.30	0.22 ± 0.30	0.24 ± 0.30	0.014	0.002	0.834

*P_a_*, medium GS group compared with low GS group; *P_b_*, high GS group compared with low GS group; *P_c_*, the high GS group compared with the medium GS group.

### Baseline data of DELC and non-DELC in CHD group

The study focused on patients diagnosed with CHD and conducted subgroup analysis based on the presence of DELC, classifying CHD patients into a DELC group (*n* = 818) and a non-DELC cohort (*n* = 253). The results demonstrated that individuals in the DELC group exhibited a significantly greater age compared to those in the non-DELC group (62.99 ± 8.29 vs. 55.26 ± 10.70, *P* < 0.001). The levels of APO-B, CysC and DM in the DELC group were higher with respect to the non-DELC group (*P* < 0.05). (Table 3).

**Table 3:** Characteristics of the CHD participants with and without DELC.

Variables	Without DELC	DELC	*t/χ^2^*	*P* value
	(*n* = 253)	(*n* = 818)		
Age (years)	55.26 ± 10.70	62.99 ± 8.29	−12.053	0.000
Male	143 (56.50)	510 (62.30)	2.756	0.097
Hypertension	166 (65.60)	544 (66.50)	0.069	0.793
DM	59 (23.30)	263 (32.20)	7.168	0.007
Smoking	125 (49.40)	395 (48.30)	0.097	0.756
TG (mmol/L)	2.34 ± 2.04	1.92 ± 1.42	3.605	0.000
AIP	0.25 ± 0.32	0.17 ± 0.29	3.788	0.000
APO-B (g/L)	1.03 ± 0.90	1.39 ± 0.43	3.559	0.000
CysC (mg/L)	0.97 ± 0.23	1.03 ± 0.21	−3.854	0.000

### Logistic regression was employed to investigate the determinants of CHD

**Figure 3:**
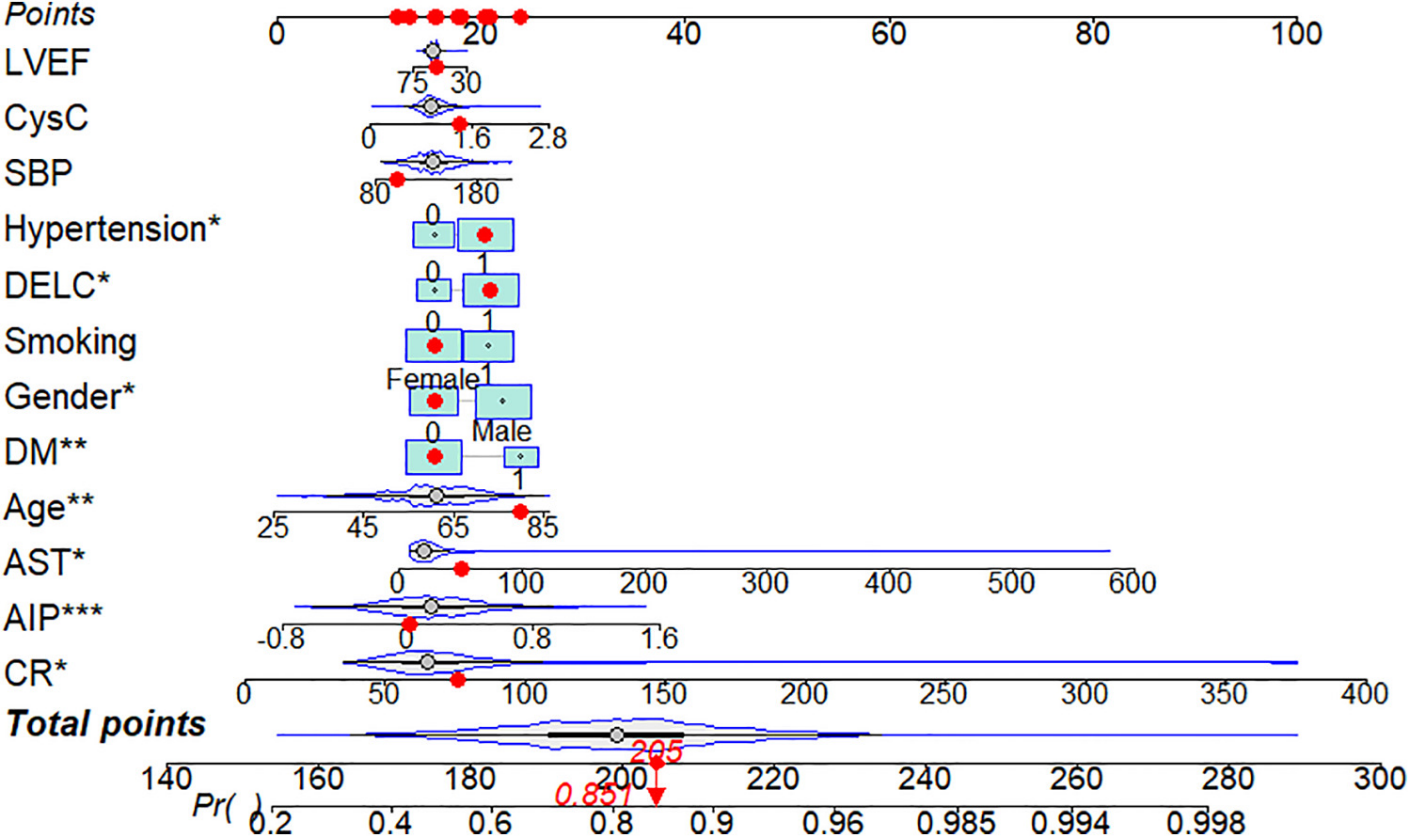
Nomogram of DELC combined with AIP.

**Figure 4:**
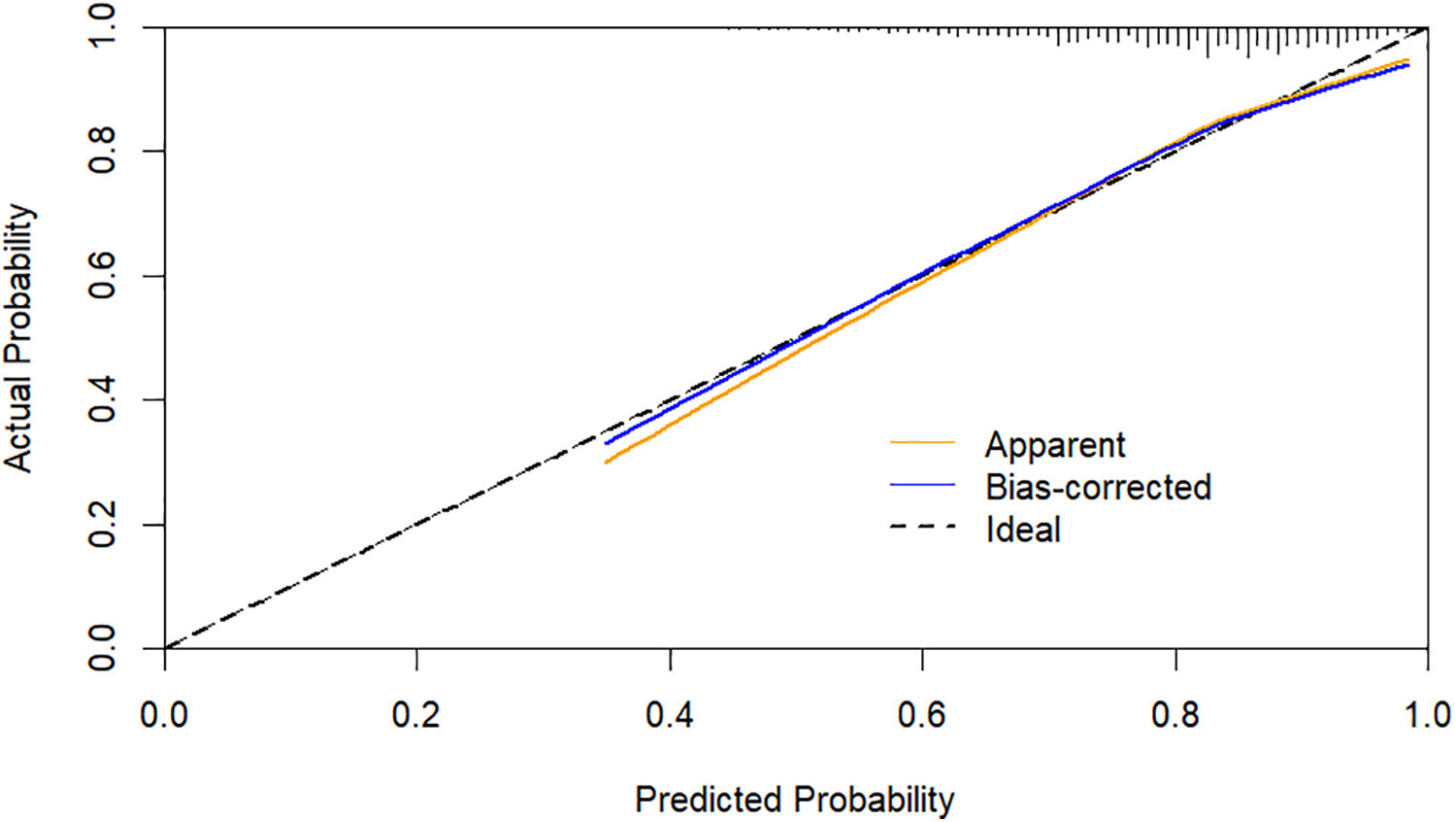
Calibration curves for DELC combined with AIP.

**Figure 5:**
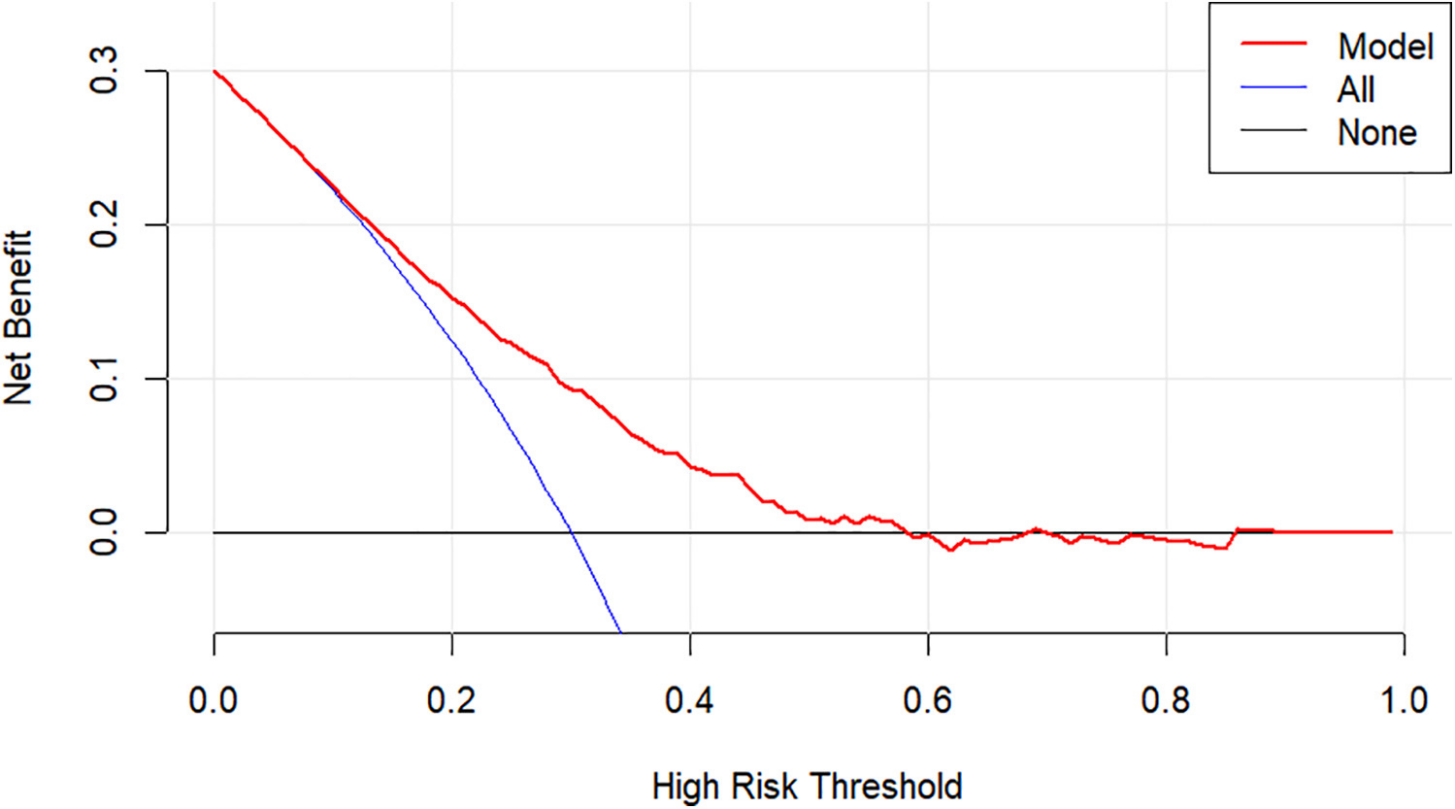
Decision curve for DELC combined with AIP.

Using CHD status (0 = No, 1 = Yes) as the dependent variable, we implemented a multivariate logistic regression analysis with age, gender, hypertension, DM, smoking, SBP, LVEF, AST, CR, AIP, CysC, and DELC as independent variables. The results indicate that age, gender, hypertension, DM, AST, CR, AIP, and DELC are independent risk factors for CHD (*P* < 0.05). Visualizing a multivariate Logistic regression model by plotting corresponding nomogram can provide the predicted probability of CHD risk. In the figure, age, AST, CR, AIP, CysC are the actual measured values, and the rest are dichotomous variables. The Hosmer-Lemeshow test was 0.614. In this study, an 80-year-old female patient (40 points) with a documented history of hypertension (21 points), no history of diabetes (15 points) and smoking (15 points), presented with a SBP of 102 mmHg (12 points), a LVEF of 55% (16 points), an AST of 50 U/L (18 points), a CR of 76 umol/L (17 points), an AIP of 0.01 (11 points), a CysC of 1.39 mg/L (18 points), and a DELC positive (22 points). The total score for this patient was 205 points, which corresponds to a CHD risk of 0.851. The calibration curve revealed that the Hosmer-Lemeshow test statistic was 0.614, indicating a strong concordance between the predicted probabilities and the observed outcomes. Furthermore, a decision curve analysis was undertaken to evaluate the model's efficacy in facilitating clinical decision-making, demonstrating that the model provides substantial clinical benefit within a threshold probability range of 0.1–0.6. After adjusting for the aforementioned risk factors, the combination of AIP and DELC emerges as a noteworthy risk factor for CHD (OR = 1.923, 95% CI: 1.095–3.378, *P* = 0.023) (Table 4, Figures 3–5).

**Table 4:** The univariate and multivariate logistic analysis of CHD by DELC combined with AIP.

Variables	Univariate analysis		Multivariate analysis	
	OR (95% CI)	*P* value	OR (95% CI)	*P* value
Age(years)	1.025 (1.012, 1.039)	0.000	1.027 (1.011, 1.043)	0.001
Gender (male = 1, female = 0)	2.099 (1.623, 2.715)	0.000	1.578 (1.084, 2.296)	0.017
Hypertension (yes = 1, no = 0)	1.671 (1.291, 2.162)	0.000	1.395 (1.045, 1.861)	0.024
DM (yes = 1, no = 0)	1.927 (1.402, 2.648)	0.000	1.773 (1.262, 2.490)	0.000
Smoking (yes = 1, no = 0)	1.925 (1.474,2.513)	0.000	1.383 (0.969, 1.974)	0.074
SBP (mmHg)	1.009 (1.002, 1.015)	0.010	1.007 (0.999, 1.014)	0.081
LVEF (%)	0.963 (0.937, 0.990)	0.007	0.990 (0.960, 1.020)	0.507
AST (U/L)	1.009 (1.002,1.016)	0.012	1.008 (1.001, 1.015)	0.021
CR (umol/L)	1.032 (1.022, 1.042)	0.000	1.016 (1.002, 1.030)	0.020
AIP	2.784 (1.776, 4.362)	0.000	2.581 (1.579, 4.219)	0.000
CysC (mg/L)	7.097 (3.441, 14.639)	0.000	1.653 (0.658, 4.154)	0.285
DELC (yes = 1, no = 0)	1.857 (1.415, 2.437)	0.000	1.405 (1.029, 1.920)	0.033
DELC + AIP	2.755 (1.630, 4.656)	0.000	1.923 (1.095, 3.378)	0.023

### Subgroup analysis

Subgroup analysis was undertaken based on age (<65 years or ≥65 years), sex, hypertension, DM, and smoking, with interaction effects assessed using *P*-values. The results indicated no significant interaction between the aforementioned variables. This suggests that AIP and DELC are independently associated with the risk of CHD, and this association remains relatively consistent across different subgroups (*P* for interaction > 0.05). (Supplementary Material S1).

### The predictive ability of AIP, DELC, and their combined use for forecasting the onset of CHD

**Figure 6:**
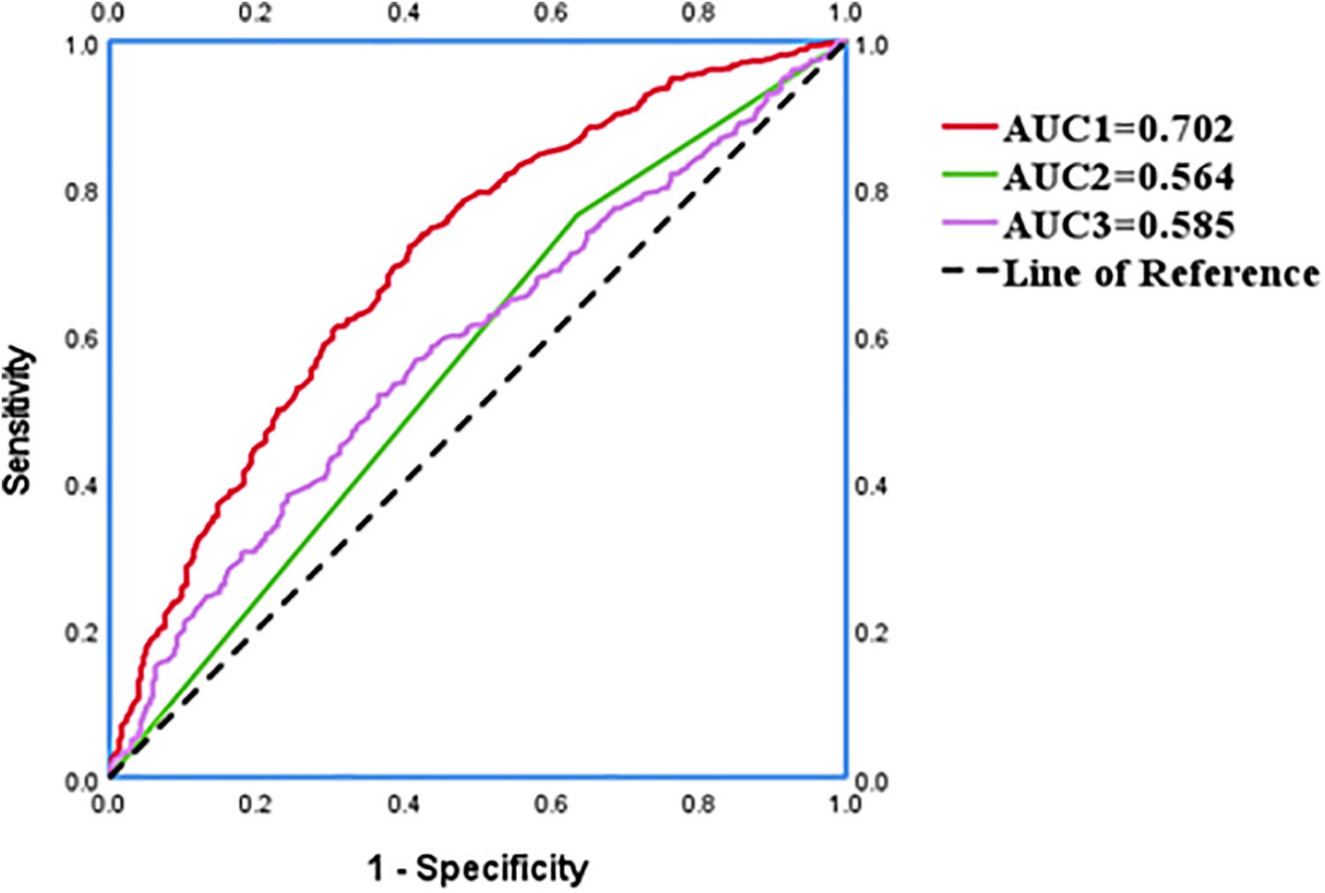
An ROC analysis with respect to the detection of CHD.

The ROC curve was generated to appraise AIP and DELC in the prediction of CHD. For AIP, the AUC was 0.585 (95% CI: 0.550–0.620), with an optimal cut-off value identified at 0.16. At this threshold, the sensitivity was 51.90%, and the specificity was 63.50%, indicating that AIP >0.16 was helpful to predict the occurrence of CHD. The AUC for DELC in forecasting the occurrence of CHD was 0.564 (95% CI: 0.527–0.602). The AUC1 of AIP and DELC combined to predict CHD was 0.702 (95% CI: 0.668–0.735), the sensitivity was 73.80%, and the specificity was 57.70%. The VIF of each independent risk factor was less than 10, and there was no multicollinearity. As shown in the figure, the combined predictive value of AIP and DELC exceeded the single predictive value (*P* < 0.05). The Hosmer-Lemeshow test yielded a value of 0.388, hinting a strong concordance between the predicted probabilities and the observed outcomes (Figure 6).

## Discussion

CHD is intrinsically an atherosclerotic disorder characterized by pervasive inflammatory processes, which may manifest clinically in various forms. The 2023 report from the European Society of Cardiology indicates that cardiovascular diseases are responsible for approximately 3 million deaths annually, with CHD accounting for the largest proportion of these fatalities (27). Additionally, the 2023 edition of Report on Cardiovascular Health and Disease in China highlights a concerning, continuous increase in CHD-related mortality from 2012–2021 (28). These alarming trends highlight the urgent need to identify reliable biomarkers and diagnostic indicators that can facilitate the early detection and timely intervention of CHD, ultimately enhancing patient outcomes.

CHD emerges from the intricate interplay of genetic predispositions and environmental influences, with atherosclerosis serving as the primary pathophysiological mechanism (3). Dyslipidemia assumes a pivotal position in the initiation of subendocardial atherogenesis and the subsequent formation of plaques within the coronary arteries. Dobiásová et al. (29) were the pioneers in introducing the concept of the AIP, a novel metric derived from the logarithmic ratio of TG vs. HDL-C. AIP functions as a valuable biomarker, encapsulating the relationship between atherosclerotic processes and the protective role of lipoproteins. In a comprehensive study conducted in Korea, which encompassed 1,124 participants undergoing multiple assessments of coronary artery calcification (CAC) via multislice computed tomography (CT), a significant correlation was established between AIP levels and the progression of CAC among individuals devoid of cardiovascular disease (CVD). The findings indicated a positive association, revealing that CAC levels escalated in tandem with elevated AIP values (30). A separate investigation conducted in 2018 examined a cohort of 348 postmenopausal women diagnosed with CHD, compared to a control group of 348 individuals. This study uncovered markedly higher AIP levels within the CHD population. After meticulous adjustment for various clinical confounders, the analysis affirmed AIP as an independent risk factor associated with the development of CHD (OR = 3.619, 95% CI: 2.003–6.538) (31). Moreover, a hospital-based observational study focusing on individuals under the age of 35 who underwent CAG revealed that AIP was independently linked to the occurrence of acute coronary syndrome (ACS). This investigation illustrated a progressive increase in both the incidence of ACS and Gensini scores in conjunction with rising AIP levels (32). Our correlation analysis further substantiated these observations. An AIP range of 0.1–0.24 has been documented to signify moderate cardiac risk (33). The ROC curve analysis within this study established an optimal cutoff value of 0.16 for AIP, indicating an elevated risk for the onset of CHD when AIP levels exceed this threshold.

The discovery and subsequent naming of DELC can be credited to Frank in 1973; however, its historical antecedents traced back to the era of Roman Emperor Hadrian (76–138 AD), who was believed to have succumbed to heart-related complications. Notably, busts and sculptures of Hadrian prominently display bilateral DELC, providing early evidence of its clinical relevance (34). In addition, DELC had been observed in the portrait of Cardinal Ludovico Trevisan, who succumbed to heart failure in 15th-century Italy (35). Ancient Chinese medical practitioners referred to this anatomical feature as the “coronary groove,” positing its potential link to coronary atherosclerosis (36). According to traditional acupuncture body maps, the earlobe crease is anatomically aligned with the heart region of the auricle, further reinforcing its possible cardiovascular implications (37). Pathological investigations have indicated a substantial association between morphological alterations in the myocardium and the presence of earlobe folds, alongside elastic fibrosis in arterial smooth muscle, Wallerian degeneration of peripheral nerves, and deep tissue fibrosis at the fold's base (38). Research indicated that the presence of coronary CHD was linked to a deficiency in the subdermal matrix, characterized by diminished elasticity and the wrinkling of the earlobes (18). Kaukola et al. (39) reported a DELC positivity rate of 72% in the CHD group (*P* < 0.001), underscoring a notable relationship between DELC and CHD. Furthermore, the positivity rate was found to increase with advancing age and the severity of the condition. This study further elucidated that DELC functions as an independent risk factor contributing to the onset of CHD, exhibiting a significantly elevated positivity rate in the CHD cohort in comparison with the non-CHD group (76.40% vs. 63.50%, *P* < 0.001). Subgroup analysis of the CHD group revealed that individuals with DELC were significantly older compared to those without DELC.

In this investigation, we found that the positivity rate of AIP levels and DELC in the CHD cohort were elevated in the CHD cohort compared to the control cohort. Further analysis revealed that both AIP and DELC serve as independent risk factors in the occurrence of CHD (OR = 1.584, 95% CI: 1.145–2.191; OR = 1.403, 95% CI: 1.027–1.916;). The AUC for the combined prediction of CHD occurrence using AIP and DELC was 0.702, which significantly exceeded the predictive values of each factor alone. This combination yielded a specificity of 57.70% and a sensitivity of 73.80%. Therefore, the integration of AIP and DELC exhibits considerable predictive value for CHD.

The present study has the following advantages: Firstly, to the best of our knowledge, this is the inaugural study to integrate AIP and DELC for predicting CHD. The integration of these two indicators in this study exhibited a substantial enhancement in the predictive performance for CHD, surpassing the diagnostic value of either indicator when utilized individually. This finding suggests that the combination of AIP and DELC possesses an advanced predictive capability for CHD. Moreover, in comparison with conventional CHD assessment strategies (CAG), this integrated approach offers a superior level of convenience and efficiency, accompanied by a substantial reduction in financial expenditures. Finally, by adjusting for potential confounding factors such as age, gender, smoking history, diabetes, and hypertension, we have enhanced the robustness and reliability of our findings. A potential limitation of this study is that AIP levels are susceptible to interference from dietary factors and lipid-lowering drug therapy, which may affect the interpretation of the study results. In addition, it was executed as a single-center investigation with a geographically constrained sample, necessitating the incorporation of external data in future research substantiate the model's predictive capabilities. Therefore, it is imperative to acknowledge the potential influence of lifestyle factors on the pathogenesis of CHD.

## Conclusions

Both AIP and DELC serve as independent prognostic markers for CHD. When considered in combination, they demonstrate a robust predictive capacity for the onset of CHD, offering a valuable tool for assessing risk, mitigating the likelihood of missed diagnoses, and enhancing overall prognostic accuracy.

## Data Availability

The raw data supporting the conclusions of this article will be made available by the authors, without undue reservation.
