# Correction: Predictive value of atherogenic index of plasma in combination with diagonal earlobe crease in coronary heart disease

**DOI:** 10.3389/fcvm.2025.1681217

**Published:** 2025-09-05

**Authors:** Ruoling Guo, Mingliang Sun, Huihui Yang, Jie Dou, Jie Gao, Tong Liu, Fei Cheng, Donglei Luo

**Affiliations:** ^1^ Department of Chengde Medical University, Chengde, Hebei, People’s Republic of China; ^2^ Department of Emergency, Handan City First Hospital, Handan, Hebei, People’s Republic of China; ^3^ Department of Tianjin Key Laboratory of Ionic-Molecular Function of Cardiovascular Disease, Department of Cardiology, Tianjin Institute of Cardiology, Second Hospital of Tianjin Medical University, Tianjin, People’s Republic of China; ^4^ Department of Neurology, Chengde Central Hospital/Second Clinical College of Chengde Medical University, Chengde, Hebei, China; ^5^ Department of Cardiology, Chengde Central Hospital/Second Clinical College of Chengde Medical University, Chengde, Hebei, China

**Keywords:** coronary heart disease, atherogenic index of plasma, diagonal earlobe crease, predict, model

There was a mistake in Figure 1 as published. The sample size for the groups was incorrect. The corrected Figure 1 appears below.

**Figure 1:**
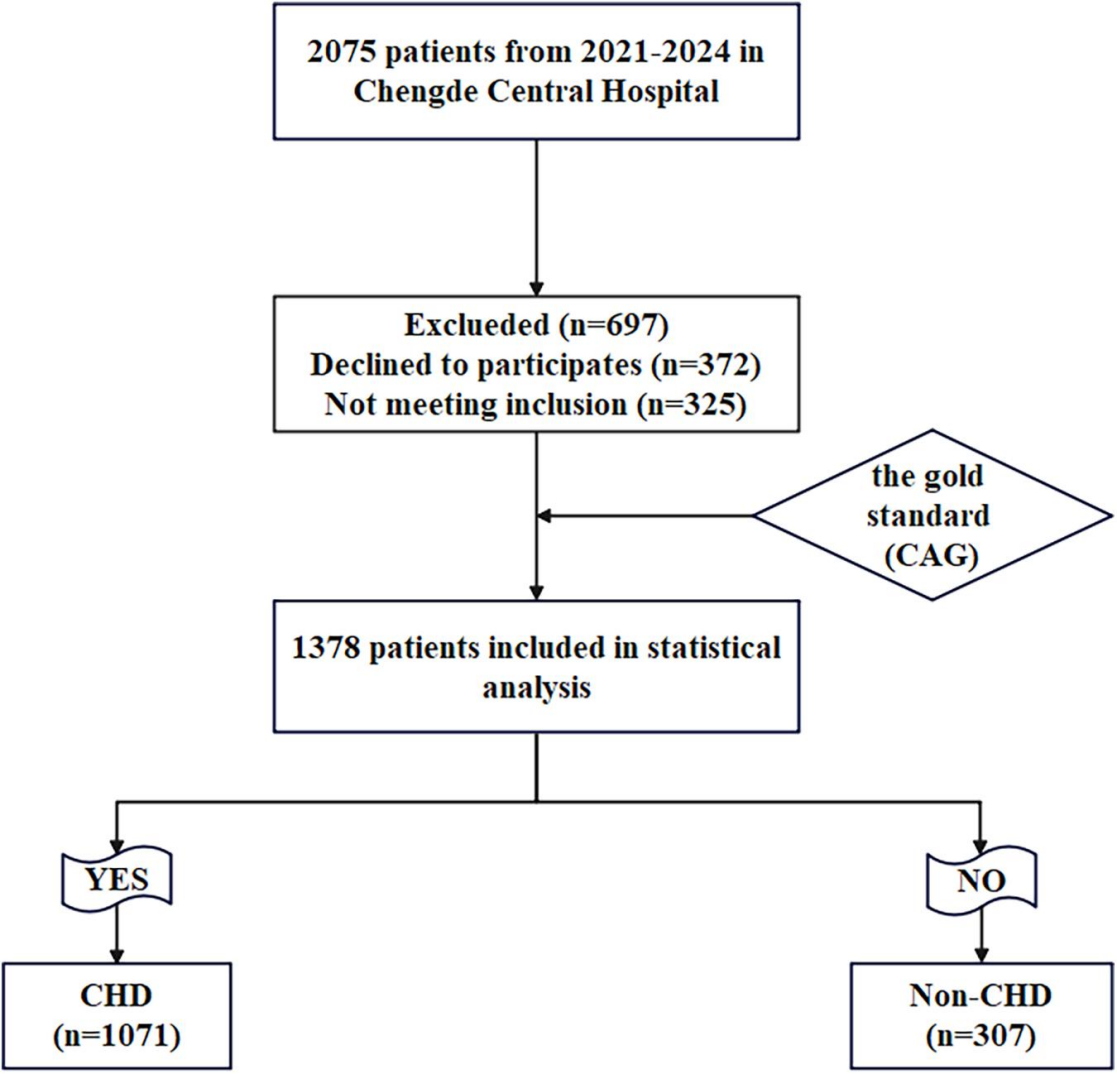
Flow-diagram illustrating patient flow in the study.

The original version of this article has been updated.

