# Supplementary figures and images for: Predictive value of atherogenic index of plasma in combination with diagonal earlobe crease in coronary heart disease
Source: Front Cardiovasc Med. 2025 Aug 6;12:1632009. doi: 10.3389/fcvm.2025.1632009 (PMC12364857; doi:10.3389/fcvm.2025.1632009)

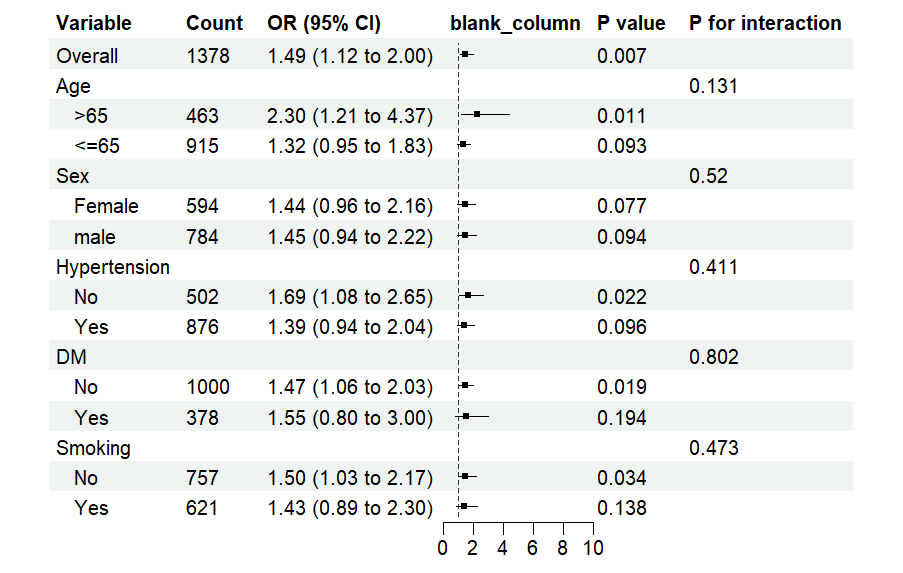

Supplement: Supplementary file 1 [file Image1.tiff]
